# Suicidal Behaviour, Self-Harm and Related Factors: A Retrospective Study from the Adolescent Psychiatric Unit

**DOI:** 10.3390/children13010147

**Published:** 2026-01-20

**Authors:** Sigita Lesinskienė, Miglė Zabarauskaitė, Tadas Valiulis, Giedrius Dailidė, Arūnas Germanavičius

**Affiliations:** Clinic of Psychiatry, Institute of Clinical Medicine, Faculty of Medicine, Vilnius University, 01513 Vilnius, Lithuania; migle.zabarauskaite@mf.stud.vu.lt (M.Z.); tadas.valiulis@mf.stud.vu.lt (T.V.); giedrius.dailide@rvpl.lt (G.D.); arunas.germanavicius@mf.vu.lt (A.G.)

**Keywords:** children, adolescents, self-harm, suicide attempts, psychosocial factors, inpatient unit

## Abstract

***Background and objectives***: Suicide attempts and self-harm are critical issues in adolescence, often leading to serious and irreversible consequences. These behaviours frequently co-occur and share common biopsychosocial risk factors. Identifying these factors enables a more comprehensive assessment of suicide and self-harm risk, helping specialists recognize high-risk individuals and implement effective preventive measures. This study aimed to examine the association between suicide attempts, self-harm and psychosocial factors among hospitalized adolescents. ***Materials and methods***: A retrospective data analysis was performed using the database of the University Department of Children and Adolescents of the Republican Vilnius Psychiatric Hospital. The study covered patients’ records from December 2022 to February 2025. Information on gender, age, suicide attempts, self-harm, adverse events (bullying, psychological abuse, physical violence within the family, and sexual abuse) and unhealthy habits (smoking, harmful alcohol consumption, and psychoactive substance use), was selected and analyzed in this study. A Chi-square test was used to assess the difference between groups. Results were considered statistically significant when *p* < 0.05. ***Results***: The study included 599 hospitalized adolescents (26.9% boys; mean age 15.1 ± 1.4 years), of whom 70.8% reported at least one episode of self-harm and 37.8% at least one suicide attempt. Rates of self-harm and suicide attempts were significantly higher in girls than in boys (self-harm: 81.3% vs. 42.2%, ϕ=0.381, p<0.001; suicide attempts: 45.5% vs. 16.5%, ϕ=0.304, p<0.001), and adolescents with self-harm had a significantly higher prevalence of suicide attempts than those without self-harm (46.7% vs. 15.8%, ϕ=0.308, p<0.001). Adverse childhood experiences and unhealthy behaviours were significantly more frequent in adolescents with self-harm and suicide attempts, although effect sizes were small to moderate (ϕ range 0.086–0.230, all p<0.05). In multivariable models, female gender (β=0.355, p<0.001) and smoking (β=0.330, p<0.001) were the strongest predictors of self-harm, whereas alcohol use (β=0.337, p<0.001) and self-harm (β=0.232, p<0.001). ***Conclusions***: Exposure to adverse childhood experiences and engagement in unhealthy habits were associated with higher rates of both self-harm and suicide attempts. A comprehensive assessment and early detection of self-harm behaviours and adverse psychosocial circumstances are crucial elements of effective suicide prevention strategies and prompt intervention among high-risk adolescents.

## 1. Introduction

Suicide remains one of the most pressing public health challenges of the 21st century. According to the World Health Organization (WHO), over 720,000 people die by suicide each year. In 2019, suicide accounted for 1.3% of all global deaths [1]. Although global suicide rates have shown a gradual decline in recent years, suicide continues to represent a particularly severe public health concern in Lithuania. National data from the Institute of Hygiene (IH) indicate that suicide accounted for 1.72% of all deaths in Lithuania, exceeding the global average and placing the country among those with the highest suicide rates in Europe [2].

The burden of suicide is especially pronounced among young people. WHO data from 2024 show that suicide was the third leading cause of death among individuals aged 15–29 worldwide [3]. In Lithuania, suicide and suicidal behaviour among adolescents and young adults remain a critical concern, underscoring the urgent need for locally adapted, evidence-based suicide prevention strategies. Although international research on this topic is extensive, empirical data focusing on contemporary Lithuanian youth, particularly in relation to psychosocial risk factors, remains limited.

Suicidal behaviour includes not only completed suicide but also suicidal thoughts, planning, attempts, and non-suicidal self-injury (NSSI) [4]. Unlike suicide attempts, self-harm involves deliberately injuring one’s own body tissue without conscious suicidal intent [5]. However, growing theoretical and empirical evidence suggests that NSSI and suicide attempts are closely interrelated. NSSI is often used as a maladaptive coping strategy to regulate overwhelming emotions, psychological distress, or to respond to adverse interpersonal experiences. Engagement in NSSI has been shown to increase the risk of subsequent suicide attempts. According to WHO, there are approximately 20 suicide attempts for every completed suicide [6]. This relationship is partly explained by shared risk factors, including trauma, violence, social isolation, mental illnesses such as depression, and sociodemographic characteristics. Sociodemographic factors such as age, gender, family structure, and socioeconomic status may further influence vulnerability to NSSI and suicidal behaviour, yet these variables remain underexplored in the Lithuanian context. While self-harm is not intended to cause death, its presence significantly elevates the risk of suicidal behaviour and attempts [7].

Understanding broader factors related to suicide can help clarify which psychosocial conditions should be prioritized in prevention and intervention efforts. Previous suicide attempts and self-harm often represent only the tip of the iceberg, while interventions aimed solely at directly reducing self-harm in adolescents have shown limited or inconsistent effectiveness [8,9]. This highlights the need for interventions that target the underlying causes of suicidal behaviour rather than focusing exclusively on its manifestations. Therefore, it is important to examine a broad range of additional factors that may contribute to suicidal behaviour, such as childhood abuse, bullying, adverse life events, or substance use [10].

Despite extensive international research, empirical population-based data from Lithuania examining non-suicidal self-injury and suicide attempts in relation to multiple psychosocial and sociodemographic factors remain limited. In Lithuania, suicide and suicidal behaviour among adolescents and young adults therefore continue to represent a critical public health concern, underscoring the need for locally adapted, evidence-based prevention strategies. The present study aims to address this gap by examining the prevalence of non-suicidal self-injury and suicide attempts and their associations with psychosocial factors among adolescents and young adults in present-day Lithuania. Specifically, this study seeks to identify key psychosocial and sociodemographic factors associated with suicidal behaviour, thereby contributing novel, locally relevant evidence to the existing literature and informing more effective prevention and intervention efforts.

## 2. Materials and Methods

The aim of this study was to comprehensively assess suicide attempts and self-harm among adolescents, including relevant biopsychosocial factors, in order to identify common associations between these phenomena and to examine their clinical significance.

To achieve this aim, a retrospective study was chosen to analyze cases of suicide attempts and self-harm documented in clinical documentation, which is especially suitable for studying important clinical phenomena. Moreover, this design permits the examination of related biopsychosocial factors utilizing routinely collected clinical data, while circumventing the ethical and logistical constraints associated with prospective or experimental studies involving high-risk adolescent groups.

Retrospective analysis was conducted using the medical records database of the University Department of Children and Adolescents at the Republican Vilnius Psychiatric Hospital (hereinafter referred to as RVPL UVPS). Data on patients treated within the department from December 2022 to February 2025 (inclusive) were collected retrospectively. The ward from which patient data were included in the study is providing care for adolescents with severe psychiatric disorders requiring hospitalization. All eligible hospitalizations within the specified timeframe were included and analyzed independently by a single individual.

Information was obtained regarding age, gender, self-harm, suicide attempts, adverse childhood experiences, including bullying, physical and psychological abuse, and sexual abuse, and harmful habits—smoking, alcohol consumption, and psychoactive substance use. All data originated solely from medical records, encompassing both clinically observed details and self-reports by the adolescent and/or their caregivers, documented by healthcare providers. Self-harm (non-suicidal self-injury) was defined as intentional injury without suicidal intent, such as cutting, burning, or hitting oneself, as recorded in the medical files. Suicide attempts referred to any self-harm with suicidal intent, documented through clinical assessment or reports from the patient or caregiver. Bullying was characterized as repeated peer harassment or aggression reported by the adolescent or caregivers and recorded in the medical history. Psychological abuse involved emotional harm (e.g., humiliation or intimidation), physical abuse involved bodily harm, and sexual abuse encompassed any sexual assault or exploitation, as documented in the records. Events not mentioned in the records were considered absent. No additional self-report tools were employed.

All analyzed patients were younger than 18 years old. Only patients whose medical records contained comprehensive data on all biopsychosocial factors within the database were included in this study; no other exclusion criteria were applied. Statistical analyses were performed using R-Commander software. To assess lifetime occurrence and recurrence, self-harm and suicide attempts were recorded using a three-point scale: 0 indicating absence, 1 representing a single instance, and 2 denoting multiple episodes. Harmful behaviours such as smoking, alcohol consumption, and psychoactive substance use were similarly rated on a three-point scale: 0 for absence, 1 for experimental or single use, and 2 for repeated use or dependence. All other variables, including adverse childhood experiences, were recorded dichotomously, with 0 signifying absence and 1 signifying presence. These numerical codes were subsequently converted into categorical labels for analytical purposes.

All statistical analyses for this study were conducted utilizing SPSS version 29. A cross-tabulation table was used to examine the relationships between variables, with the Chi-square test (*χ*^2^) employed to assess statistical significance and corresponding effect sizes calculated to evaluate the strength of associations. Spearman’s rank correlation test was used to assess the relationships between numerical and ranked variables. A linear regression model was used to examine the association between the dependent variable and one or more independent variables while accounting for potential confounding factors. To ensure the model’s appropriateness for drawing valid conclusions, several key criteria were evaluated: a coefficient of determination (R^2^) exceeding 0.20 and an ANOVA *p*-value less than 0.05. Statistical significance was defined as *p* < 0.05.

Permission to use the data for scientific purposes was obtained from the hospital (No. K1-183), as well as approval from the Bioethics Committee (No. (1.7E) 150000-KT-454).

## 3. Results

The study included 599 patients: 161 boys (26.9%) and 438 girls (73.1%). The average age was 15.1 years (SD = 1.4), with ages ranging from 10 to 17 years at the time of hospitalization.

### 3.1. Self-Harm and Suicide Attempts

In total, 70.8% (n = 424) of patients had experienced at least one episode of self-harm. 37.8% (n = 224) had attempted suicide at least once. The prevalence of self-harm and suicide attempts in the sample is presented in Table 1 and Table 2.

The study results showed a statistically significant association between suicide attempts and self-harm (Pearson $\chi^{2}$ = 49.41, *p* < 0.001), with effect size estimates (*Φ* = 0.308) indicating a moderate-strength association. Among hospitalized adolescents who had engaged in at least one episode of self-harm, 46.7% (n = 197) had also attempted suicide at least once, versus 15.8% (n = 27) among those without self-harm. Also, a statistically significant but weak correlation was identified between self-harm and the number of suicide attempts (ρ_s_ = 0.315, *p* < 0.001).

The study results showed a statistically significant association between gender and self-harm (Pearson *χ*^2^ = 86.77, *p* < 0.001), with effect size estimates (*Φ* = 0.381) indicating a moderate-strength association. 81.3% (n = 356) of female participants reported experiencing at least one episode of self-harm, while among male participants, 42.2% (n = 68). A similar statistically significant relationship has been identified between gender and the incidence of suicide attempts (Pearson *χ*^2^ = 41.64, *p* < 0.001). The effect size estimates (*Φ* = 0.304) indicate a moderate-strength association. 45.5% (n = 198) of the female respondents indicated that they had attempted suicide at least once, versus 16.5% (n = 26) of male respondents reported a suicide attempt.

### 3.2. Adverse Experience, Self-Harm, and Suicidal Attempts

56.5% (n = 338) of patients had experienced bullying, 42.3% (n = 253) psychological abuse, physical violence within the family context, 37.0% (n = 221), and 18.7% (n = 112) sexual violence. The prevalence of adverse experience in the sample is presented in Table 3.

Pearson’s chi-squared test (*χ*^2^) revealed that patients who reported at least one episode of self-harm in their lifetime had a significantly higher prevalence of bullying experiences compared to those who did not engage in self-harm (61.3% vs. 44.8%). The results showed a Pearson *χ*^2^ value of 13.65, with a *p* < 0.001. The effect size estimate (*Φ* = 0.151) suggests a weak association between self-harm and experiences of bullying. Similar observations were made, in which patients who self-harmed were statistically more likely to have experienced psychological abuse and physical violence within the family context than those who never self-harmed (46.2% vs. 32.8%; 40.8% vs. 27.6%) (*χ*^2^ = 9.16, *p* = 0.002; *χ*^2^ = 9.24, *p* = 0.002). However, a weak association was found in both cases (*Φ* = 0.124). Patients who self-harmed were significantly more likely to have experienced sexual abuse than those who did not (21.5% vs. 12.1%, *χ*^2^ = 7.152, *p* = 0.004). However, the effect size (*Φ* = 0.109) suggests a weak association between self-harm and sexual abuse. More comprehensive comparisons of adverse childhood experiences and self-harm are examined in Table 4.

Pearson’s chi-squared test (*χ*^2^) revealed that patients reporting suicidal behaviour had a higher prevalence of bullying experiences compared to those who did not (62.5% vs. 53.1%), with a *χ*^2^ value of 4.99 and *p* = 0.025. The effect size (*Φ* = 0.092) indicates a very weak association between suicide attempts and bullying. Similar findings were noted for psychological abuse and physical violence; patients with suicidal behaviour had higher rates of these experiences (53.6% vs. 36.0% and 48.2% vs. 30.4%, respectively) with *χ*^2^ values of 17.50 and 19.05 (*p* < 0.001). However, the associations remained weak (*Φ* = 0.172 and *Φ* = 0.179). Additionally, patients who attempted suicide were more likely to have experienced sexual abuse (23.2% vs. 16.3%, *χ*^2^ = 4.40, *p* = 0.038), though the effect size (*Φ* = 0.086) suggests a very weak association. Further details on adverse childhood experiences and suicide attempts are provided in Table 5.

### 3.3. Unhealthy Habits, Self-Harm, and Suicide Attempts

38.2% (n = 229) of patients reported smoking occasionally, while 14.4% (n = 86) were nicotine dependent. Additionally, 40.1% (n = 240) used alcohol excessively, with 6.8% (n = 41) being dependent on it. Furthermore, 25.0% (n = 150) had experimented with drugs, and 9.5% (n = 57) were addicted.

Patients who reported self-harm in their lifetime had a significantly higher prevalence of being dependent on nicotine or smoking occasionally than those who did not self-harm at all (16.3% vs. 9.7%; 42.0% vs. 29.1%, *χ*^2^ = 18.88, *p* < 0.001), but the effect size showed a weak association between smoking and self-harm (Cramer’s V = 0.178). We found similar trends, in that patients who self-harmed were significantly more likely to use alcohol or try psychoactive substances harmfully and to be dependent on alcohol or drugs (*χ*^2^ = 8.06, *p* = 0.017; *χ*^2^ = 8.59, *p* = 0.012), but the effect size showed a weak association between alcohol, psychoactive substances and self-harm (Cramer’s V = 0.116; Cramer’s V = 0.122). Table 6 provides a more detailed comparison of unhealthy habits and self-harm.

Patients with a history of suicide attempts were more likely to report occasional smoking (52.2% vs. 29.8%, *χ*^2^ = 31.36, *p* < 0.001) but less likely to report nicotine addiction (8.9% vs. 17.9%, *χ*^2^ = 31.36, *p* < 0.001), compared with patients without a history of suicide attempt. However, the corresponding effect size suggested a weak association between suicide attempt and smoking (Cramer’s V = 0.230). Similarly, individuals who had attempted suicide were more likely to report harmful alcohol use or drug experimentation (*χ*^2^ = 15.54, *p* < 0.001; *χ*^2^ = 9.60, *p* = 0.008), although the effect sizes indicated weak associations in both cases (Cramer’s V = 0.162; Cramer’s V = 0.127). Further details on unhealthy habits and suicide attempts are provided in Table 7.

### 3.4. Predictors of Self-Harming Behaviour and Suicide Attempts

A linear regression analysis was conducted to predict self-harming behaviour and suicide attempts among patients based on adverse childhood experiences, unhealthy habits, gender, and history of suicide attempts (F = 31.306, *p* < 0.001, R^2^ = 0.257), (F = 75.960, *p* < 0.001, R^2^ = 0.213). The results indicated that female gender (*β* = 0.355, *p* < 0.001) and smoking (*β* = 0.330, *p* < 0.001) were the strongest predictors of self-harming behaviour among patients. Meanwhile, the strongest predictors of suicide attempts were alcohol consumption (*β* = 0.337, *p* < 0.001) and self-harm behaviour (*β* = 0.232, *p* < 0.001). However, it was observed that smoking acts as a negative predictor of suicide attempts (*β* = −0.186, *p* < 0.001). The negative beta coefficient for smoking warrants cautious interpretation due to the use of three categorical groups with heterogeneous distributions across key covariates. This non-homogeneity likely introduces residual confounding, and the finding should not be taken as evidence of a protective effect. Table 8 presents the full linear regression analysis.

## 4. Discussion

The present study adds to the growing body of international evidence demonstrating a strong association between self-harm and suicide attempts among adolescents. Data from the RVPL UVPS study indicate that adolescents with a history of self-harm were nearly three times more likely to report a suicide attempt compared to their peers without such a history. Similar patterns have been observed across different cultural and geographical contexts, indicating that the association between self-harm and suicide attempts is consistently reported across studies. For example, Can Shao et al. reported that suicide attempts were almost five times more prevalent among Chinese adolescents who had engaged in self-harm, while meta-analysis by Donna Gillies et al., encompassing nearly 600,000 adolescents, found that suicide attempts were nine times more likely among individuals with a history of self-harm [11,12]. Taken together, these findings suggest that NSSI should be understood not merely as an isolated or transient behaviour, but as a clinically meaningful indicator associated with elevated vulnerability to suicidal behaviour. From a theoretical perspective, self-harm may reflect maladaptive strategies for regulating intense emotional distress or coping with adverse interpersonal experiences [13].

The present findings further align with longitudinal research indicating that a history of self-harm is associated with a higher likelihood of subsequent suicidal behaviour. According to Stephanie M.Y. Wong et al., the likelihood of suicide within the subsequent 12 months is 1.71 times higher among individuals who have engaged in self-harm [14]. In clinical terms, these findings underscore the importance of recognizing self-harm as a significant prognostic marker of suicide risk. While a single episode of self-harm does not necessarily imply suicidal intent, recurrent or persistent self-harm may warrant closer monitoring and comprehensive risk assessment in both clinical and school-based mental health settings.

Gender emerged as a significant sociodemographic factor associated with both self-harm and suicide attempts in the RVPL UVPS study. Consistent with international evidence, girls were more likely than boys to report self-harm and suicide attempts. Similar gender differences have been documented in large population-based studies and meta-analyses, for instance a 2024 United Kingdom study by Emma Diggins et al., involving over 11,000 fourteen-year-olds, reported that self-harm was nearly three times more prevalent among girls than boys [15]. Similarly, a 2022 meta-analysis of 25 studies by Yu-Jing Wang and colleagues found that the likelihood of self-harm among girls was three times greater than among boys [16]. According to RVPL UVPS data, girls engaged in self-harm almost twice as frequently as boys and were 2.7 times more likely to have attempted suicide. These patterns are corroborated by other studies, such as Claudio A. Dávila Cervantes et al., which found that girls were over three times more likely to attempt suicide [17]. This gender disparity is often attributed to increased social pressures related to appearance, behaviour, and relationships, leading to heightened emotional distress among girls, who may consequently resort to self-harm or suicide attempts as coping mechanisms [18]. While women are more frequently affected by self-harm and suicide attempts globally, men are significantly more likely to die by suicide, often on their first attempt [19]. Together, these findings highlight the need for prevention and intervention approaches that are sensitive to gender-specific risk profiles and expressions of psychological distress.

Exposure to childhood trauma, including bullying as well as psychological, physical, and sexual violence, was also strongly associated with self-harm and suicide attempts in the present study. These results are consistent with international research demonstrating that adolescents exposed to bullying or violence are more likely to report self-harm and suicidal behaviour. For example, Hui Chen et al. surveyed over 1300 Chinese students aged 12–16 and found that bullying was associated with nearly four times higher prevalence of self-harm and a three times higher prevalence of suicide attempts [20]. Comparable findings were reported by Ingri Myklestad et al. in a Norwegian study involving over 14,000 students, where bullying was linked to five times higher prevalence of self-harm [21].

Notably, psychological violence showed a particularly strong association with these outcomes, a finding that aligns with large-scale studies indicating that non-physical forms of violence may have a profound impact on adolescent emotional well-being. The RVPL UVPS data revealed an association between psychological violence exposure and self-harm in adolescents. These findings are supported by a 2024 study by Yitong He et al., which analyzed data from over 95,000 Chinese adolescents and found that self-harm was 1.26 times more common among those who experienced psychological violence [22]. Further, suicide attempts were 1.55 times more frequent among those reporting psychological abuse. Similar trends were identified in a 2019 study by Maciej Zygo et al., which observed that psychological abuse was 2.3 times more common among adolescents who had attempted suicide compared to those who had not [23]. Additionally, research by Abigail J. Lyons et al. in 2025 demonstrated that adolescents who experienced psychological abuse from parents were three times more likely to attempt suicide within the following year, compared to their non-abused peers [24].

Physical violence, like psychological violence, was significantly associated with self-harm and suicide attempts. A 2021 study by Emmanuel Nii-Boye Quarshie et al., involving over 2000 adolescents, found that exposure to physical violence remained associated with self-harm after adjusting for other adverse experiences [25]. The RVPL UVPS data revealed associations between physical violence exposure and both self-harm and suicide attempts in adolescents. Consistent findings were reported by Maciej Zygo et al. in Poland, where students with a history of physical violence were 2.5 times more likely to have attempted suicide than those without such experiences [23].

Comparable patterns are evident regarding the impact of sexual abuse. A 2024 meta-analysis by Natalia Calvo et al., synthesizing findings from 46 studies, demonstrated that individuals with a history of sexual abuse were 2.7 times more likely to engage in self-harm [26]. Similarly, a 2020 meta-analysis by I. Angelakis and colleagues, drawing on 79 studies, found that the likelihood of attempting suicide was 3.5 times higher among those who had experienced sexual violence [27]. These results align with data from the RVPL UVPS study and underscore the critical role of sexual abuse as a potent risk factor for self-harm and suicide.

Harmful habits, including smoking, alcohol consumption, and the use of psychoactive substances, were likewise associated with higher prevalence of self-harm and suicide attempts. For instance, David Lawrence et al. found that 32% of adolescent smokers reported self-harming, compared to only 10% of non-smokers [28]. These findings suggest that smoking may serve as a maladaptive coping mechanism for stress. Similarly, Phil H. Lee and colleagues observed that adolescents who smoked were three to five times more likely to attempt suicide than their non-smoking peers [29]. The association between alcohol use and suicidality is also well documented: Claudio A. Dávila-Cervantes reported that adolescents who consumed alcohol were 1.7 times more likely to attempt suicide [17], while Christina M. Sellers demonstrated that this likelihood increased to 2.7 times even before psychiatric hospitalization [30]. Collectively, these findings underscore the necessity of assessing harmful substance use as a significant predictor of suicide risk in clinical practice.

While self-harm was observed among adolescents regardless of psychoactive substance use, its prevalence was significantly higher among those who used or were addicted to psychoactive substances. Early initiation of psychoactive substance use has been associated with long-term alterations in brain regions involved in emotional regulation and impulse control, which may increase vulnerability to self-harm [31]. Supporting this, Alexander Denissoff et al. found that cannabis use before ages 15–16 was associated with a fourfold increase in the risk of self-harm in adulthood [32]. Although the present data do not allow for conclusions regarding causality, the strong associations observed highlight substance use as an important clinical marker that should be routinely assessed in suicide risk evaluations.

Several limitations of the present study should be acknowledged. The cross-sectional design limits the ability to determine the temporal ordering of self-harm, psychosocial risk factors, and suicide attempts, and the reliance on self-reported data may introduce recall or reporting biases. In addition, the study did not differentiate between adolescents who engaged in single versus repetitive episodes of self-harm or suicide attempts, despite evidence suggesting that these patterns may represent clinically distinct subgroups. Notably, only four adolescents within the current sample qualified as high-volume repeaters—defined as having been hospitalized fifteen or more times for the same reason—which hindered the possibility of conducting meaningful subgroup analyses.

Recent research employing novel methodological approaches has shown that a small proportion of self-harm patients—referred to as high-volume repeaters—account for a disproportionately large share of self-harm-related healthcare attendances, despite representing a very small percentage of the overall clinical population [33]. Future research in Lithuanian samples could benefit from examining such patterns in greater detail, as this may improve risk stratification and support the development of more targeted prevention and intervention strategies.

Overall, the present findings contribute to the growing literature on adolescent self-harm and suicidal behaviour by providing contextually relevant evidence from Lithuania and serve as a basis for further research in the field and the cultural comparisons. By highlighting the associations between self-harm, psychosocial stressors, harmful habits, and sociodemographic factors, this study underscores the importance of comprehensive, multi-dimensional approaches to suicide prevention that extend beyond symptom-focused interventions and address the broader psychosocial context of adolescent mental health.

## 5. Conclusions

This retrospective study demonstrated an association between self-harm and suicide attempts among adolescents hospitalized in a psychiatric unit. Adolescents with a history of self-harm were significantly more likely to have attempted suicide, underscoring self-harm as a key clinical marker of elevated suicide risk. Pronounced gender differences were observed, with girls showing higher rates of both self-harm and suicide attempts, indicating the need for gender-sensitive assessment and prevention strategies.

Exposure to adverse childhood experiences, including bullying and psychological, physical, and sexual abuse, was consistently associated with increased prevalence of self-harm and suicide attempts. Unhealthy habits, such as harmful alcohol use and psychoactive substance use, were also linked to higher risk, highlighting the importance of integrated assessment of psychosocial adversity and substance-related behaviours. The unexpectedly lower rate of suicide attempts among adolescents with nicotine dependence suggests a complex relationship between substance use patterns and suicidal behaviour that warrants further investigation. Overall, effective suicide prevention in high-risk adolescents requires comprehensive, trauma-informed, and developmentally tailored interventions.

## Figures and Tables

**Table 1:** Distribution of patients according to recurrence of self-harm.

Recurrence of Self-Harm	Percent of Patients (%)	Number of Patients (n=)
No self-harm	29.2	175
First-time self-harm	2.5	15
History of self-harm episode	11.5	69
Repeated self-harm	56.8	340

**Table 2:** Distribution of patients by suicide attempts.

Recurrence of Attempted Suicide	Percent of Patients (%)	Number of Patients (n=)
Did not attempt suicide	62.2	369
Single suicide attempt	16.2	96
Multiple suicide attempts	21.6	128

**Table 3:** Prevalence of Adverse Childhood Experiences Among Patients.

Adverse Childhood Experience	Percent of Patients (%)	Number of Patients (n)
Bullying	56.5	338
Psychological abuse	42.3	253
Physical abuse	37.0	221
Sexual abuse	18.7	112

**Table 4:** Comparison of Adverse Childhood Experiences with Patients’ Self-Harm.

	Self-Harm						
**variables**	yes		no		*χ* ^2^	*p*	*Φ*
	n	%	n	%			
**Bullying**							
yes	260	61.3	78	44.8	13.65	<0.001	0.151
no	164	38.7	96	55.2			
**Psychological abuse**							
yes	196	46.2	57	32.8	9.16	0.002	0.124
no	228	53.8	117	67.2			
**Physical abuse**							
yes	173	40.8	48	27.6	9.24	0.002	0.124
no	251	59.2	126	72.4			
**Sexual abuse**							
yes	91	21.5	21	12.1	7.152	0.004	0.109
no	333	78.5	153	87.9			

**Table 5:** Comparison of Adverse Childhood Experiences with Patients’ Suicide Attempts.

	Suicide Attempt						
**variables**	yes		no		*χ* ^2^	*p*	*Φ*
	n	%	n	%			
**Bullying**							
yes	140	62.5	196	53.1	4.99	0.025	0.092
no	84	37.5	173	46.9			
**Psychological abuse**							
yes	120	53.6	133	36.0	17.50	<0.001	0.172
no	104	46.4	236	64.0			
**Physical abuse**							
yes	108	48.2	112	30.4	19.05	<0.001	0.179
no	116	51.8	257	69.6			
**Sexual abuse**							
yes	52	23.2	60	16.3	4.40	0.038	0.086
no	172	76.8	309	83.7			

**Table 6:** Comparison of unhealthy habits and patients’ self-harm.

	Self-Harm						
**variables**	yes		no		*χ* ^2^	*p*	Cramer’s V
	n	%	n	%			
**Smoking**					18.88	<0.001	0.178
no	177	41.7	107	61.1			
occasionally	177	42.0	51	29.1			
dependence	69	16.3	17	9.7			
**Alcohol**					8.06	0.017	0.116
no	210	49.5	108	61.7			
occasionally	185	43.6	55	31.4			
dependence	29	6.8	12	6.9			
**Psychoactive substances**					8.59	0.012	0.122
no	262	61.8	130	74.3			
occasionally	118	27.8	32	18.3			
dependence	44	10.4	13	7.4			

**Table 7:** Comparison of unhealthy habits and patients’ suicide attempts.

	Suicide Attempts						
**variables**	yes		no		*χ* ^2^	*p*	Cramer’s V
	n	%	n	%			
**Smoking**					31.36	<0.001	0.230
no	87	38.8	193	52.3			
occasionally	117	52.2	110	29.8			
dependence	20	8.9	66	17.9			
**Alcohol**					15.54	<0.001	0.162
no	97	43.3	216	58.6			
occasionally	113	50.4	126	34.1			
dependence	14	6.3	27	7.3			
**Psychoactive substances**					9.60	0.008	0.127
no	131	58.5	256	69.4			
occasionally	72	32.1	77	20.9			
dependence	21	9.4	36	9.8			

**Table 8:** Linear Regression Analysis Predicting Self-Harming Behaviour and Suicide Attempts Among Patients.

Variable	Self-Harm			Suicide Attempts		
	B	SE_B_	*β*	B	SE_B_	*β*
Intercept	−0.449	0.105		0.198	0.200	
Gender	0.364	0.041	0.355 *	0.339	0.081	0.183 *
Bullying	0.090	0.034	0.098 *	0.074	0.065	0.045
Psychological abuse	−0.055	0.042	−0.060	0.226	0.078	0.136 *
Physical abuse	0.016	0.040	0.017	0.105	0.076	0.062
Sexual abuse	0.025	0.044	0.022	−0.110	0.083	−0.052
Smoking	0.209	0.038	0.330 *	−0.213	0.073	−0.186 *
Alcohol	−0.102	0.049	−0.098 *	0.445	0.091	0.337 *
Psychoactive substances	0.020	0.036	0.029	−0.108	0.067	−0.088
Suicide attempts	0.118	0.021	0.214 *			
Self-harm				0.421	0.076	0.232 *

* <0.001, B = unstandardized regression coefficient, SE_B_ = standardized error of the coefficient, and *β* = standardized coefficient. Reference values: Gender = female, Bullying = yes, Psychological abuse = yes, Physical abuse = yes, Sexual abuse = yes, Smoking = addicted, Alcohol = addicted, Psychoactive substances = addicted, Suicide attempts = multiple suicide attempts, Self-harm = at least one episode of self-harm.

## Data Availability

The original contributions presented in this study are included in the article. Further inquiries can be directed to the corresponding authors.
